# Empathy Modulates the Effects of Acute Stress on Anxious Appearance and Social Behavior in Social Anxiety Disorder

**DOI:** 10.3389/fpsyt.2022.875750

**Published:** 2022-07-13

**Authors:** Bernadette von Dawans, Amalie Trueg, Marisol Voncken, Isabel Dziobek, Clemens Kirschbaum, Gregor Domes, Markus Heinrichs

**Affiliations:** ^1^Department of Biological and Clinical Psychology, University of Trier, Trier, Germany; ^2^Department of Psychology, Biological Psychology, Clinical Psychology and Psychotherapy, University of Freiburg, Freiburg, Germany; ^3^Department of Psychology and Neuroscience, Clinical Psychological Science, Maastricht University, Maastricht, Netherlands; ^4^Clinical Psychology of Social Interaction, Humboldt-Universität zu Berlin, Berlin, Germany; ^5^Biological Psychology, Technical University Dresden, Dresden, Germany

**Keywords:** social anxiety disorder (SAD), stress, empathy, cortisol, TSST-G

## Abstract

Patients suffering from social anxiety disorder (SAD) fear social interaction and evaluation, which severely undermines their everyday life. There is evidence of increased prosocial behavior after acute social stress exposure in healthy individuals, which may be interpreted as stress-regulating “*tend-and-befriend”* behavior. In a randomized controlled trial, we measured empathic abilities in a first diagnostic session. In the following experimental session, we investigated how patients with SAD (*n* = 60) and healthy control participants (HC) (*n* = 52) respond to an acute social stressor (Trier Social Stress Test for groups) or a non-stressful control condition, and whether empathic abilities and acute social stress interact to modulate anxious appearance and social behavior in a social conversation test. Salivary cortisol, heart rate, and subjective stress response were repeatedly measured. The anxious appearance and social behavior of participants were rated by the conversation partner. SAD patients demonstrated stronger subjective stress responses while the biological responses did not differ from HC. Moreover, patients performed worse overall in the conversation task, which stress additionally undermined. Finally, we found that both emotional and cognitive empathy buffered the negative effects of acute stress on social behavior in SAD, but not in HC. Our data highlight the importance of empathic abilities for SAD during stressful situations and call for multimodal clinical diagnostics. This may help to differentiate clinical subtypes and offer better-tailored treatment for patients.

**General Scientific Summary:** This study shows that high levels of cognitive and emotional empathy can buffer the negative effects of acute stress on social behavior in social anxiety disorder (SAD). Empathic abilities may be included as an additional diagnostic resource marker for SAD.

## Introduction

Although stress is well known for its’ negative consequences on physical and mental health (1, 2), there is mixed evidence on the behavioral effects of acute stress leading to either antisocial or prosocial behavior [for an overview see (3)]. There is strong evidence that specific situational aspects and personality variables influence the actual response to acute stressors (4). Empathic abilities playing a crucial role in social interactions may be one of these modulators. Understanding and feeling the other’s emotional and mental state, i.e., empathy, is one of our most fundamental abilities underlying social interaction. As such, empathy is associated with affiliation and prosocial behavior and is a prerequisite for smooth (pro-) social interactions (5–8). Empathy may be divided into its cognitive and emotional elements. While cognitive empathy is defined as the ability to recognize the emotion another person is feeling, emotional empathy reveals how strongly one feels the other person’s emotion (9). Despite socio-cognitive abilities like empathy being a prerequisite for successful social interactions, little is known about the role that empathic abilities play in modulating social behavior during psychosocial stress exposure.

Moreover, the role of various psychopathologies within the link between stress and social behavior is still not well understood. Patients suffering from social anxiety disorder (SAD) in particular can differ from healthy controls in how they react to social stress. Behavioral responses to stress may be characterized in two directions (antisocial or prosocial), and are referred to evolutionarily concepts of “fight-or-flight” or “tend-and-befriend” (10, 11). It is important to mention that prosocial behavioral reactions to stressful situations are certainly not possible (or suitable) in every situation. But if possible and when appropriate, they are potent regulatory acts modulating our own emotions and feelings of stress, as well as in strengthening our social skills and social bonds in the future (12, 13). However, for patients suffering from SAD such social interactions are the focus of their fears. SAD is characterized by the distinct fear of behaving embarrassingly when interacting with others. That is, social interactions imply marked distress for these patients. In reaction, they exhibit what is known as avoidance behavior (14). Moreover, there is accumulating evidence that SAD patients exhibit actual deficits in social performance (15–17) and that other detect these impairments rapidly (17–20). This raises the question of whether tend-and-befriend responses to stress are even feasible for patients with SAD.

Although social evaluative situations constitute the key fear for SAD, there is still inconclusive evidence for an exaggerated psychobiological stress response to social evaluative stress in SAD. The literature has documented exaggerated fear responses in the amygdala (21) as well as hyper-reactivity of the hypothalamus-pituitary-adrenal axis (HPA) to social stress like the Trier Social Stress Test (TSST) (22). But the experimental findings have been inconsistent and point toward discordance between subjective and physiological stress responses in SAD (23), thus leaving us with an unclear impression about differences in the psychobiological stress response to social stress in SAD compared to healthy controls (HC).

A few studies have tested the effects of acute stress on social behavior in SAD considering the effects of acute stress on social behavior. Mallott et al. (24) compared healthy participants with high and low social anxiety scores, respectively. Subjects with high social anxiety levels acted less prosocially after social rejection than participants low in social anxiety. To date, only one study (25) examined the influence of stress on social behavior in a clinical sample of SAD patients. Subjects with SAD avoided angry faces faster after stress in an approach-avoidance task, indicating increased avoidance behavior. At the same time it is well-known that SAD patients exhibit an attentional bias for threat [(26); for a review on neuroimaging data see (27)]. That is, SAD patients are hypervigilant to negative and threatening stimuli. Stress impairs the prefrontal cortex’s function, thereby impeding working memory and the regulation of attention in favor of “bottom up” control [for reviews see (28, 29)]. Thus, under acute stress, SAD patients’ bias for threat may be even more pronounced, facilitating withdrawal and impeding social approach and prosocial behavior. In accordance, patients with SAD showed increased processing of threatening faces after cortisol administration compared to placebo (30). There is also evidence that in SAD, attention under stress is quite self-focused and the processing of external cues is reduced (31, 32). Most notably, none of the aforementioned studies examined social cognitive abilities *ex ante*. Moreover, as no study has examined genuine “face to face” social interactions, any conclusions about SAD patients’ actual behavior in real-life social situations are limited.

What remains unclear about social anxiety is whether empathic abilities are better, worse or the same as HC. A recent meta-analysis (33) describes an overall association between emotional empathy and social anxiety but no association between cognitive empathy and social anxiety. In their subgroup analyses they report a slightly negative association with lower cognitive empathy in SAD. The authors discuss the “great variability between studies” and call for additional research. We thus planned our study to test not only the direct effects of acute psychosocial stress on social interactive behavior during a real face to face conversation in SAD compared to healthy controls (HC). We also chose a paradigm that resembles a naturalistic two person, face-to-face conversation with a rating of “anxious appearance” and “social behavior” by the interaction partner as basic variables of social interaction (17). Moreover, we wanted to clarify the role that empathic abilities play within this relationship, resembling another basic competence for social interactive behavior. We hypothesized that (I) SAD patients would show deficits in social performance compared to HC, irrespective of stress. Further, it was expected that (II) in healthy individuals, acute stress would lead to affiliative behavior in terms of increased social performance and that (III) SAD patients would not exhibit affiliative behavior in reaction to stress but instead would be characterized by intensified social deficits. Finally, we expected that (IV) empathic abilities would modulate these adverse effects of stress on social performance.

## Materials and Methods

### Participants

All our participants were recruited via advertisements in local newspapers, notices and flyers. Online questionnaires and telephone interviews were used to check eligibility. Exclusion criteria were age under 18 years or over 55 years, any acute or chronic medical condition including neurological disorders, smoking of more than five cigarettes per day, current use of medication, drug or alcohol abuse, a body mass index (BMI) > 30, or shiftwork. Participants could also not participate if they were students of psychology or economics and if they were not naïve to the TSST procedure. Other exclusion criteria for the SAD group were any other current mental disorder except SAD or avoidant personality disorder. For the control group, participants could not have or have had any mental disorder diagnosis. Due to sex hormone influences on the cortisol stress response, we included only male participants (34). We based our sample size calculation on previous studies with similar designs (35, 36) and planned to recruit 30 participants per group including possible drop-outs or missing data. A total of 121 subjects (63 SAD; 58 HC) met these criteria and participated in our experiment. From this sample, three subjects with SAD and six healthy controls had to be excluded for this reasons: current medication use (*n* = 5), critically elevated global psychiatric symptoms in control sample [*t*-value of the Brief Symptom Inventory > 70; (37)] (*n* = 3), one subject was known to one of the experimenters (*n* = 1). Our final sample consisted of 112 participants, with 60 SADs, age 28.30 ± 8.91 years (mean ± *SD*), and 52 healthy controls, age 27.85 ± 6.67 years. Participants were randomly assigned to the stress or no-stress condition of the Trier Social Stress Test for groups (TSST-G (38); SAD: 31 in the stress condition, 29 in the no-stress condition; healthy controls: 27 in the stress condition, 25 in the no-stress condition).

### Empathic Abilities

The Multifaceted Empathy Test [MET; (39)] was used to measure emotional and cognitive empathic abilities. It consists of 30 pictures showing people in positive and negative emotionally laden situations. To assess cognitive empathy, participants had to choose the correct mental state description out of four labels presented at the bottom of the picture. To measure emotional empathy, participants rated their experienced level of empathic concern for the person in the picture on a 9-point Likert scale. Trial presentation and response registration was controlled by a PC running Presentation (Neurobehavioral Systems Inc., Albany, CA, United States). For statistical analyses, trials within the cognitive or emotional condition were averaged to provide us with one dependent variable. Due to technical problems, the sample is missing MET data from five participants.

### Psychosocial Stress Manipulation

For the stress manipulation, we applied the TSST-G, a standardized laboratory protocol inducing psychosocial stress in groups of up to six participants (38). In the TSST-G, subjects are separated by dividing walls to prevent them from interacting. The TSST-G comprises two conditions, namely, a stress and a control (no-stress) condition. In the stress condition, participants have to give a 2-min free speech for a mock job interview and do a mental arithmetic task, both in front of two evaluators and two cameras. In the no-stress condition, participants had to read a text in a low voice and recite number series and are not videotaped or evaluated in any way. This resembles an active control condition with the same physiological features (standing upright, talking) but without the stress-inducing aspects of social evaluation or uncontrollability that are specific to the stress condition. The TSST-G enables a moderate psychosocial stress induction and has been proven to reliably result in activation of the HPA axis with elevated cortisol levels, as well as significant cardiovascular and subjective stress responses with a specific and active control condition that does not lead to psychobiological stress responses (38, 40, 41).

### Stress Response Measures

**FIGURE 1:**
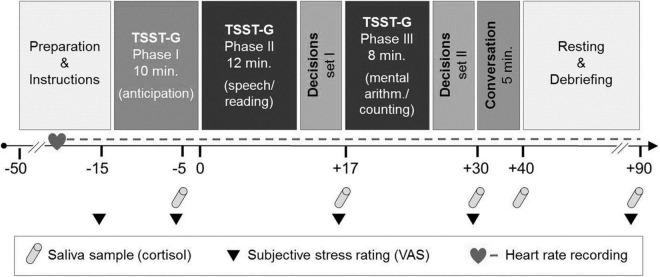
Experimental course with stress induction and the social conversation paradigm [adapted from von Dawans et al. (35)]. TSST-G, Trier Social Stress Test for Groups. VAS, visual analogue scale.

We measured salivary cortisol and heart rate to assess the physiological stress response. To measure the subjective stress reaction, a visual analogue scale (VAS) was used. Cortisol and subjective responses were collected at five time points throughout the experiment (Figure 1).

Saliva samples for cortisol assaying were collected using a commercially available sampling device (Sarstedt^®^, Nümbrecht-Rommelsdorf, Germany). Saliva samples were stored at −20°C. For biochemical analyses of free cortisol concentration, saliva samples were thawed and spun at 3.000 revolutions per minute for 10 min to obtain 0.5–1.0 ml of clear saliva of low viscosity. Salivary cortisol concentrations were determined by a commercially available chemiluminescence immunoassay (CLIA; IBL, Hamburg, Germany). Inter- and intra-assay coefficients of variation were below 8%. Because of too little saliva, one participant’s cortisol data were not available (*n* = 111).

Heart rate was measured via a wireless chest heart rate transmitter and wrist monitor recorder (Polar RS800TM, Polar Electro^®^, Oy, Kempele, Finland) and recorded continuously as an indicator of the sympathetic adrenal medullary system (SAM) (see experimental course, Figure 1). We assessed an aggregated baseline mean over 5 min in a standing position. For the stress induction period, means from 35 1-min intervals were entered into the analyses. Due to technical problems, heart rate measures were not available for *n* = 11 at the baseline and *n* = 8 participants during the stress induction period. Heart rate analyses were thus conducted with *n* = 101 participants at baseline and *n* = 104 during the stress induction period.

### Social Interaction Paradigm: Social Performance

We assessed social performance via an everyday conversation task, based on Voncken and Bögels (17). This paradigm enables an experimentally controlled investigation of social interaction with high ecological validity. Social performance comprises social behavior and anxious appearance (see below). Participants had to start a 5-min conversation with a confederate. Instructions were as follows: “We would like you to have a conversation with another person. The purpose is to get to know each other. It is up to you to start the conversation and to keep it going.”

Confederates were female undergraduate students. All had undergone a 3 h training session in the experimental conversation paradigm and rating social interaction with the Social Behavior and Anxious Appearance Rating Scale [SBA-rating scale; (17)]. They had been trained to remain in a neutral but friendly posture during the conversation and to answer with up to three pieces of information. They were trained to only pose a question after 7 s of silence. That way, the responsibility for keeping the conversation going remained with the participant. We based these instructions on Voncken and Bögels (17). Confederates were blinded for the condition (stress/no-stress) and group (SAD/HC). After the conversation, confederates rated the participants’ social performance via a modified version of the SBA-rating scale (17).The SBA-rating scale consists of 27 items and two scales: *anxious appearance* and *social behavior*. *Anxious appearance* covers signs of nervousness such as trembling, blushing or stuttering. Examples for the questions are: “Was the participant shaking/trembling (e.g., hands, legs or parts of the face)?” or “Was the participant laughing nervously?” *Social behavior* comprises items relating to formal aspects of interaction behavior, such as maintaining eye contact or formulating full sentences, as well as more complex aspects, such as the degree of self-disclosure or showing interest in the conversation partner. Examples for questions are: “Did the participant seem confident?” or “Did the participant make eye contact with you?” The two-factor structure was confirmed by Voncken and Bögels (17) and Bögels et al. (42). The original scale refers to a conversation involving three persons and was adapted for a two-party conversation in our study. Therefore, one item of the SBA scale had to be removed (“Could the participant divide attention between you both?”) which resulted in 26 items for the current investigation. *Social behavior* and *anxious appearance* represent the two main dependent behavioral variables of social performance in the conversation paradigm.

### Additional Psychometric Measures

To assess social anxiety as a continuous variable, we applied the Liebowitz Social Anxiety Scale [LSAS; (43)]. Depressive symptoms were rated with the Beck Depression Inventory [BDI; (44)]. To assess the general burden of psychiatric symptoms, we used the Brief Symptom Inventory [BSI; (45)]. We relied on the Perceived Stress Scale to measure chronic stress levels [PSS; (46)].

### Procedure

Participants came to the laboratory twice. The first appointment was scheduled for individual diagnostics including SKID I and SKID II [German version of the Structural Clinical Interview for DSM IV; (47)], and the Multifaceted Empathy Test [MET; (39)] for measuring cognitive and emotional empathy abilities.

After providing informed consent, all participants were interviewed by a trained clinician and performed the MET individually on a PC. The diagnostic session lasted approximately 2 h.

If participants fulfilled our inclusion criteria, they were randomly assigned to the stress or control condition. They were scheduled for the experimental session in groups of four to six participants. The groups were always mixed with participants in the SAD and HC group. Participants were asked not to do any physical exercise and to abstain from caffeine, nicotine, alcohol or any medication for 24 h prior to the experiment. They were instructed to eat their usual standard breakfast and lunch and to stop food consumption at 4 P.M. Sessions started between 5 and 6 P.M. to control for diurnal variation in cortisol secretion. After arriving at the lab, participants again provided informed consent. They were not allowed to communicate with each other, were provided with a heart rate device (Polar, RS800TM, Polar Electro^®^, Oy, Kempele, Finland) and were seated individually in a cubicle computer lab. They were given information about saliva collection and how to set markers for the heart rate recording. After filling out the first visual analogue scales, they underwent the TSST-G (38). Each of the two parts of the TSST-G (speech/reading and mental arithmetic/counting, see next section), was followed by a set of decisions on a behavioral economic paradigm [adapted from von Dawans et al. (35)]. Due to space shortage, these results are being prepared for a second manuscript. At the end of the TSST and the second decision paradigm, participants were informed that the next task would be a conversation with a stranger. They were guided individually into separate rooms where a trained female confederate was waiting and the conversation task took place for 5 min. Participants then came back into the computer lab for further questionnaires (BDI, BSI, LSAS, and PSS), saliva sampling and VAS (see Figure 1). All participants were debriefed and reimbursed at the end of the study, which was approved by the local ethics committee of the University of Freiburg, Germany. Participants gave written informed consent on both study days. Subjects received a flat fee of 40 Euros and could earn additional money in the decision paradigm (depending on their own decisions and the decisions of another group of participants not subjected to the TSST-G procedure) with a mean of 5.69 ± 0.90 Euros (*SD*).

### Statistical Analyses

To test for differences in groups with respect to psychometric data or baseline measures we conducted two factorial ANOVAs with the factors stress/control and SAD/HC. We also ran two MANOVAS, one for empathy (cognitive and emotional) and one for social performance (anxious appearance and social behavior). With respect to the moderation of the effects of stress on social behavior by empathic abilities we conducted two moderation analyses separately for each HC and SAD (one for anxious appearance, one for social behavior). The PROCESS macro for SPSS by Hayes (48) was used (model 2). Moderation analyses were conducted for both SAD and HC with condition as the independent variable, and anxious appearance and social behavior, respectively, as dependent variable; cognitive and emotional empathy as two moderators.

## Results

### Sample Characteristics

Participants with SAD and HC did not differ in age [*F*(1, 108) = 0.07, *p* = 0.786, η*_*p*_*^2^ = 0.001] or education [χ^2^ (df = 2) = 0.248, *p* = 0.883]. Participants in the stress group tended to be older [*F*(1, 108) = 3.31, *p* = 0.072, η*_*p*_*^2^ = 0.030]. As expected participants with SAD exhibited significantly higher levels of psychopathological symptoms, and they reported higher levels of social anxiety [LSAS: *F*(1, 104) = 138.56, *p* < 0.001, η*_*p*_*^2^ = 0.571], depressive symptoms [BDI: *F*(1, 107) = 60.70, *p* < 0.001, η*_*p*_*^2^ = 0.362] and general psychiatric symptoms [BSI global severity index: *F*(1, 108) = 137.79, *p* < 0.001, η*_*p*_*^2^ = 0.561] as well as higher levels of chronic stress [PSS: *F*(1, 108) = 77.84, *p* < 0.001, η*_*p*_*^2^ = 0.419]. There were neither significant differences between stress and control condition nor any significant interactions between the two factors (all *p* > 0.10) (Table 1).

**TABLE 1:** Sample characteristics.

	HC				SAD			
		
	Control		Stress		Control		Stress	
				
	*M*	*SD*	*M*	*SD*	*M*	*SD*	*M*	*SD*
Age	27.24	4.49	28.41	8.24	26.10	6.49	30.35	10.39
Education	4.2	0.58	4.19	0.68	4.24	0.51	4.26	0.73
Social anxiety (LSAS)	19.58	12.72	23.00	17.60	67.71	23.17	63.60	21.70
Depressive symptoms (BDI)	3.84	4.11	2.0	2.32	13.28	7.02	12.77	10.24
Psychiatric symptoms (BSI)	49.12	8.34	48.67	10.65	70.69	6.35	66.35	9.41
Chronic stress (PSS)	12.08	4.89	12.44	4.72	22.86	6.36	19.84	5.48

*LSAS, Liebowitz Social Anxiety Scale; BDI, Beck Depression Inventory; BSI, Brief Symptom Inventory; PSS, Perceived Stress Scale.*

Participants with SAD and healthy controls did not differ in terms of empathic abilities, in their level of cognitive or emotional empathy. We also found that randomization to the experimental conditions succeeded: there were no differences in empathic abilities between the stress and control condition and no interaction effects (all *p* > 0.10) (Table 2).

**TABLE 2:** Empathic abilities.

	HC				SAD			
		
	Control		Stress		Control		Stress	
				
	*M*	*SD*	*M*	*SD*	*M*	*SD*	*M*	*SD*
Cognitive empathy (MET)	19.61	4.00	19.96	3.43	19.54	3.10	19.69	2.41
Emotional empathy (MET	5.80	1.41	5.83	1.21	5.49	1.53	5.61	0.81

*MET, Multifaceted Empathy Test.*

### Baseline Stress Values

We noted a divergence in baseline measures between psychological stress levels and biological stress markers. Participants with SAD showed significantly higher levels on the VAS stress at baseline [*F*(1, 108) = 12.45, *p* = 0.001, η*_*p*_*^2^ = 0.103] whereas they did not differ from HC in cortisol [*F*(1, 107) = 1.35, *p* = 0.247, η*_*p*_*^2^ = 0.012] or heart rate [*F*(1, 97) = 1.81, *p* = 0.181, η*_*p*_*^2^ = 0.018] at baseline.

### Subjective Stress Response

Our within-subjects results showed that inducing stress via the TSST-G was successful. Besides an overall effect of time [*F*(3.44, 358.18) = 43.11, *p* < 0.001, η*_*p*_*^2^ = 0.293], participants in the stress condition displayed a significantly higher subjective stress response measured with the VAS stress [time × stress: *F*(3.44, 358.18) = 9.92, *p* < 0.001, η*_*p*_*^2^ = 0.087] compared to the control condition. Moreover, there was a significant time × SAD interaction documenting higher subjective responses in SAD than HC [*F*(3.44, 358.18) = 4.93, *p* = 0.001, η*_*p*_*^2^ = 0.045]. The three way interaction [time × stress × SAD: *F*(3.44, 358.18) = 2.12, *p* = 0.077, η*_*p*_*^2^ = 0.021] reached significance on the trend level.

**FIGURE 2:**
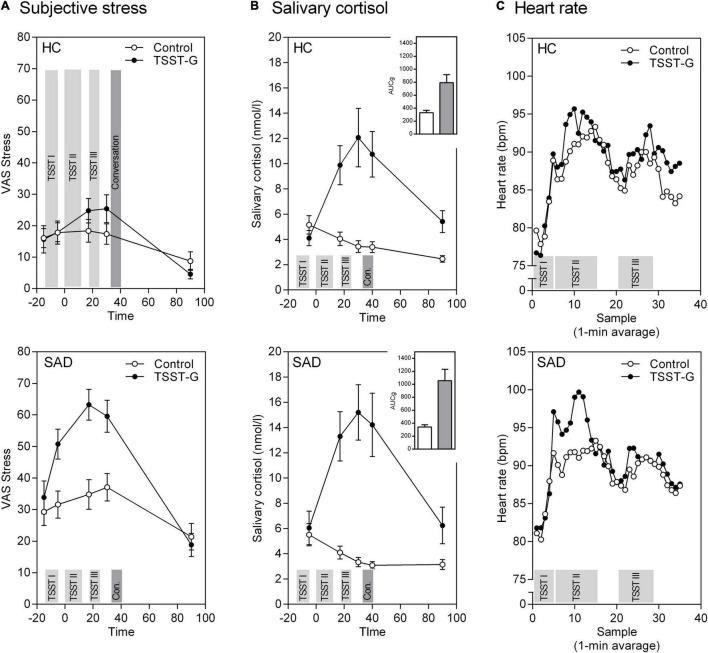
Mean (± SEM) of the psychobiological stress response in healthy controls (HC) and participants with SAD to the TSST-G and the control condition. **(A)** Subjective stress on a VAS, **(B)** free salivary cortisol concentration with Area-Under-the-Curve (AUC), and **(C)** heart rate over the course of the experiment. The experiment’s different phases are highlighted as bars: 5 min Anticipation (TSST I), Speech/Reading (TSST II), Mental arithmetic/Counting (TSST III), and Conversation.

With respect to the between-subject effects we detected significant main effects for the stress condition [*F*(1, 104) = 6.09, *p* = 0.015, η*_*p*_*^2^ = 0.055], SAD [*F*(1, 104) = 42.80, *p* < 0.001, η*_*p*_*^2^ = 0.292], and a significant interaction between stress × SAD—yet more evidence of higher overall subjective stress levels in SAD than HC [*F*(1, 104) = 4.28, *p* = 0.041, η*_*p*_*^2^ = 0.039; Figure 2A].

### Cortisol Stress Response

For the within-subjects effects, the repeated measures ANOVA for cortisol revealed an effect of time [*F*(2.21, 235.99) = 28.33, *p* < 0.001, η*_*p*_*^2^ = 0.209]. Participants in the stress condition exhibited significantly stronger cortisol stress responses than those in the control condition [time × stress: *F*(2.21, 235.99) = 39.86, *p* < 0.001, η*_*p*_*^2^ = 0.271]. The time × SAD and three way interaction time × stress × SAD were not significant. The between-subjects effects show a main effect for stress that reflects higher overall cortisol levels in the stress than in the control condition [*F*(1, 107) = 23.54, *p* < 0.001, η*_*p*_*^2^ = 0.181; Figure 2B].

The same pattern appears again concerning the areas under the curve: significant effects are revealed for stress induction for the AUC_*G*_ [*F*(1, 107) = 24.85, *p* < 0.001, η*_*p*_*^2^ = 0.188] as well as the AUC_*I*_ [*F*(1, 107) = 64.48, *p* < 0.001, η*_*p*_*^2^ = 0.376]. There were no differences in SAD vs. HC, nor any significant stress × SAD interactions (all *p* > 0.10, Figure 2B bar charts).

### Autonomic Stress Response

For heart rate responses we again found successful stress induction revealed through a significant effect of time [*F*(8.24, 824.14) = 35.64, *p* < 0.001, η*_*p*_*^2^ = 0.263] and time × stress interaction [*F*(8.24, 824.14) = 2.34, *p* = 0.016, η*_*p*_*^2^ = 0.023]. There were no other significant effects in heart rate responses to stress (Figure 2C).

Both our results on cortisol and heart rate demonstrate a divergence between subjective and biological variables that is reflected in stronger subjective responses, but similar biological responses in SAD compared to HC (Figure 2).

### Effects of Stress on Social Performance

**FIGURE 3:**
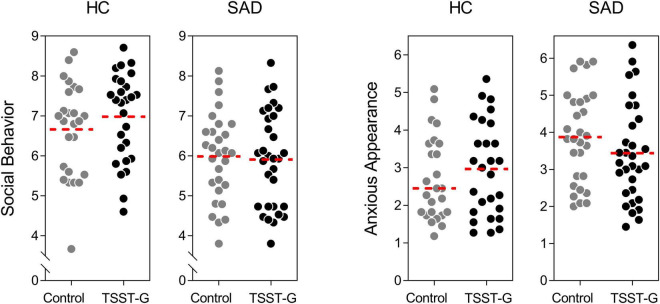
Effects of acute stress on social behavior and anxious appearance during the post-stress conversation for controls and participants with SAD; dots represent individual data, dotted lines represent the mean.

In a multivariate analysis of variance, we tested for differences in social behavior and anxious appearance in SAD compared to healthy controls, as well as for the effects of the acute stress induction. The latter failed to show a significant main effect [*F*(2, 107) = 0.161, *p* = 0.852, η*_*p*_*^2^ = 0.003, *Wilk’s*Λ = 0.997]. As expected, we observed a significantly better performance in HC than SAD [*F*(2, 107) = 8.884, *p* < 0.001, η*_*p*_*^2^ = 0.142, *Wilk’s*Λ = 0.858]. Moreover, there was a significant stress × SAD interaction revealing a different pattern for the stress induction in SAD patients vs. HC [*F*(2, 107) = 3.550, *p* = 0.032, η*_*p*_*^2^ = 0.062, *Wilk’s*Λ = 0.938; Figure 3].

### Modulation of Social Behavior Under Stress by Empathy

**FIGURE 4:**
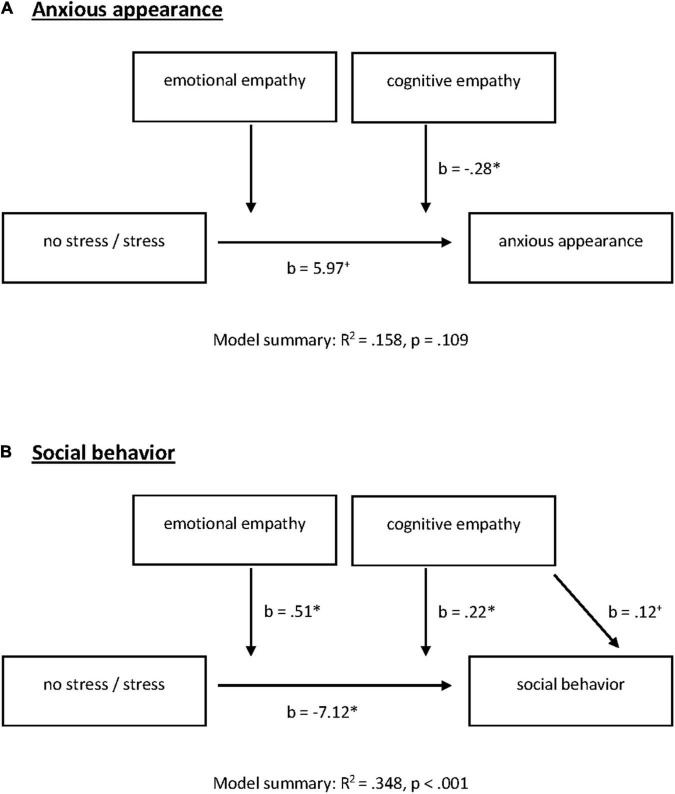
Emotional and cognitive empathy as moderators for acute stress effects on **(A)** anxious appearance and **(B)** social behavior in the post-stress conversation in participants with SAD. ^+^*p* = 0.100, *P = 0.050.

With our final step, we wanted to clarify whether empathy modulates the significant stress × SAD interaction of on social performance and used a moderation model conducted with PROCESS (Model 2) for SAD and HC separately (Figure 4).

There were no significant effects for HC. Neither stress induction nor empathic abilities nor the interaction explained anxious appearance [*F*(5, 44) = 1.18, *R*^2^ = 0.118, *p* = 0.336] or social behavior in HC [*F*(5, 44) = 1.09, *R*^2^ = 0.110, *p* = 0.382].

In SAD, the model summary for anxious appearance was not significant [*F*(5, 51) = 1.91, *R*^2^ = 0.158, *p* = 0.109]. When looking at the model, we noted a significant stress by cognitive empathy interaction [stress × cognitive empathy: *F*(1, 51) = 5.07, change *R*^2^ = 0.084, *p* = 0.029]. Moreover, stress alone predicted anxious appearance on the trend level [*b* = 5.97, *t*(51) = 1.91, *p* = 0.062; Figure 4A].

**FIGURE 5:**
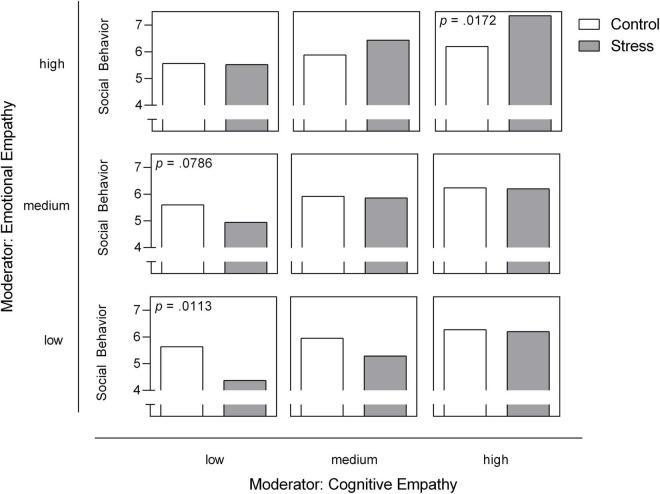
Emotional and cognitive empathy as moderators for acute stress effects on social behavior in the post-stress conversation in participants with SAD. Low, medium and high levels represent mean ± 1 *SD* (48). The level of significance for the conditional effects of the focal predictor at values of the moderators is presented in case of *p* < 0.10 and is added to the respective panel.

Our moderation model for social behavior was significant [*F*(5, 51) = 5.45, *R*^2^ = 0.348, *p* < 0.001]. We identified a significant negative effect of stress on social behavior [*b* = −7.12, *t*(51) = −3.02, *p* = 0.004]. With respect to the direct effects of empathic abilities, cognitive empathy shows significance on the trend level [*b* = 0.12, *t*(51) = 1.97, *p* = 0.055] while emotional empathy had no direct effect on social behavior (*p* = 0.803). However, both facets of empathy moderate the effects of stress on social behavior [stress × cognitive empathy: *F*(1, 51) = 5.23, change *R*^2^ = 0.067, *p* = 0.026; stress × emotional empathy: *F*(1, 51) = 4.05, change *R*^2^ = 0.052, *p* = 0.050; Figures 4B, 5]. All results of the moderation models are found in the Supplementary Material (SOM). Moreover, the figure shows the level of significance for the conditional effects of the focal predictor at values of the moderators (all parameters can be found in the SOM).

## Discussion

We tested the effects of acute stress on social behavior in HC and SAD in an experimental design, and found that empathic abilities to be a key regulator of social communication that moderate this relation.

First, our results show that SAD patients do not differ in their physiological stress responses on either the HPA or SAM—a finding in line with previous evidence showing divergent subjective and physiological stress responses (23). In our study, participants suffering from SAD again demonstrated higher overall subjective stress levels and overall stronger subjective responses. We detected neither higher heart rate or cortisol baseline levels for SAD, nor stronger physiological stress responses. Since neuroimaging studies have repeatedly documented stronger amygdala responses for SAD via fMRI (49) patients’ physiological stress systems may have been adapted from being hyper-responsive and they may sooner or later become hyporesponsive as has been documented in conjunction with other disorders (50). There is initial evidence documenting hyporeactivity of parts of the HPA axis in SAD patients following acute stress induction (51). On the other hand, it is also possible that patients with SAD may form subgroups that reveal different psychobiological reactivity patterns. It is therefore important for future studies to collect psychological and physiological reactivity data from larger samples in order to distinguish subtypes. If clinicians could more specifically characterize patients on their level of both subjective feelings and subjective responses to threatening situations (like social stressors) and measure their physiological responses, we may gain important knowledge leading to better therapeutic strategies. Patients showing hyper-responsiveness on both—subjective and physiological—levels may form a different group compared to those whose subjective and physiological responses differ (52, 53).

As expected, we found deficits in the social interactive behavior of SAD patients compared to HC, and we are able replicate findings (17). Our SAD patients were less competent in their social performance, and appeared more anxious during an everyday situation that resembled a typical “small talk” situation. Moreover, the interaction of SAD by stress demonstrates differential behavioral effects of stress in HC compared to SAD patients—results that are not surprising. Patients with SAD bear a heavy burden since they fear social interactions, an essential feature of human development and everyday regulation. But these patients are not just disadvantaged in building up relationships and negotiating their everyday routines. In HC, social stress had no effect on social performance in a face to face conversation. However, in patients with SAD, social performance continues to worsen after social stress. This is of utmost importance, because positive social interaction is such an important way to establish and seek social support and social relationships, factors that are likewise linked to both mental health and morbidity and mortality (54).

Our study integrated empathic abilities as one important diagnostic measure of social communication. Patients with SAD do not differ overall from healthy controls in their cognitive or emotional empathy level, a finding already documented by other researchers (33). But interestingly, we found that empathy buffered the detrimental effects of social stress on social behavior in SAD during the conversation paradigm. Both cognitive and emotional empathy moderated the effect of social stress on social behavior. When looking at our data in detail, note that high levels of cognitive or emotional empathy can buffer the effect of social stress on social behavior, while it is especially those patients with low empathic abilities who exhibit poorer social behavior after social stress, as empathy is known to promote other-oriented motivation and prosocial behavior (5, 7, 8). One explanation may be that high levels of empathy compensate higher levels of self-focused attention and the reduced processing of external cues under stress in SAD (31, 32). Our results again highlight the importance of expanding upon our approaches to diagnosing social anxiety disorder. As we have documented, not every patient suffering from SAD reveals impaired social communication, although standard tests may discover potential deficits or resources [e.g., the Multifaceted Empathy Test, MET (39)]. Since social communication is an important prerequisite for many psychotherapeutic approaches like exposure therapy, it is extremely important that SAD patients receive tailored treatment and training within this very domain. We can hypothesize that poor social communication is one limiting factor for cognitive behavioral therapy in SAD (55). On the other hand, patients with good to excellent social communication skills need no training and therapeutic focus can be directed toward other domains.

For both aspects, and considering diverging subjective and physiological stress reactions or fear responses, as well as social communication and how these interact, it could eventually prove to be especially worthwhile to target the brain oxytocin system. Oxytocin is known to be involved in social communication (56) and the perception of social support as well as stress regulation (40, 57, 58). However, endogenous versus exogenous oxytocin exhibit different effects depending on many individual and contextual aspects—all factors that make its use as a therapeutic drug still challenging (59). A combination of psychotherapeutic strategies and individually tailored trainings (e.g., training social cognitive skills) via oxytocin augmentation is a potentially promising future route (60–62).

The present study bears some limitations that need to be addressed in future research. First, we only tested male participants due to the influences of sex hormones on stress hormone responses (34). Our findings therefore cannot be generalized, and need to be replicated in female mixed samples. In addition, we only had one confederate interacting with one participant due to the logistic complexity of our group study. Future studies should include two confederates per interaction in order to control for the level of inter-rater reliability. Moreover, our SAD participants underwent no psychotherapeutic treatment and were not taking any psychotropic medication. Moreover, they could not have any comorbid disorder. This again resembles potential selection bias, revealing the need for future studies with clinical samples including SAD patients on treatment and with typical comorbidities so as to test our effects within a more heterogenous SAD sample. Nevertheless, our study takes an initial step toward an broadened approach to diagnostic and treatment of SAD within the social domain and will hopefully lead to further investigations of the underlying mechanisms.

Taken together, we have replicated findings on the diverging physiological vs. subjective stress responses in SAD. In addition, SAD patients demonstrated poorer social performance and a more anxious appearance in a social conversation and this effect was even pronounced after acute stress. We found that empathic abilities may buffer detrimental effects of acute stress on social performance, while empathic abilities *per se* were not lower in SAD than HC. Our findings contribute to our understanding of acute stress effects on social interaction in SAD and enhance the evidence on the importance of empathic abilities within the diagnostic process and in terms of treatment strategies.

## Data Availability Statement

The data from all experiments reported will be accessible *via* OSF doi: 10.17605/OSF.IO/TRG8M.

## Ethics Statement

The studies involving human participants were reviewed and approved by University of Freiburg, Germany. The patients/participants provided their written informed consent to participate in this study.

## Author Contributions

BD, MV, CK, ID, and MH contributed to conception and design of the study. AT and BD conducted data collection and wrote the first draft of the manuscript. BD, AT, and GD performed the statistical analysis and prepared the figures. All authors interpreted the results, contributed to manuscript revision, read, and approved the submitted version.

## Conflict of Interest

The authors declare that the research was conducted in the absence of any commercial or financial relationships that could be construed as a potential conflict of interest.

## Publisher’s Note

All claims expressed in this article are solely those of the authors and do not necessarily represent those of their affiliated organizations, or those of the publisher, the editors and the reviewers. Any product that may be evaluated in this article, or claim that may be made by its manufacturer, is not guaranteed or endorsed by the publisher.
