# Supplementary material for: Empathy Modulates the Effects of Acute Stress on Anxious Appearance and Social Behavior in Social Anxiety Disorder
Source: Front Psychiatry. 2022 Jul 13;13:875750. doi: 10.3389/fpsyt.2022.875750 (PMC9326503; doi:10.3389/fpsyt.2022.875750)
Supplement: Supplementary file 1 [file Data_Sheet_1.PDF]

# Social Behavior SAD

Run MATRIX procedure:

```
***************** PROCESS Procedure for SPSS Version 3.5 *****************

          Written by Andrew F. Hayes, Ph.D.       www.afhayes.com
    Documentation available in Hayes (2018). www.guilford.com/p/hayes3

**************************************************************************
Model  : 2
    Y  : sba_sb
    X  : cond
    W  : met_cog
    Z  : met_emo

Sample
Size:  57

**************************************************************************
OUTCOME VARIABLE:
 sba_sb

Model Summary
          R       R-sq        MSE          F        df1        df2
 p
      ,5902      ,3483      ,8870     5,4526     5,0000    51,0000      ,00
04

Model
              coeff         se          t          p       LLCI       ULCI
constant     3,8005     1,2570     3,0235      ,0039     1,2770     6,3239
cond        -7,1207     2,3610    -3,0160      ,0040   -11,8607    -2,3808
met_cog       ,1162      ,0591     1,9658      ,0548     -,0025      ,2350
Int_1         ,2169      ,0949     2,2867      ,0264      ,0265      ,4074
met_emo      -,0299      ,1194     -,2509      ,8029     -,2696      ,2097
Int_2         ,5060      ,2515     2,0117      ,0496      ,0010     1,0109

Product terms key:
 Int_1    :        cond     x        met_cog
 Int_2    :        cond     x        met_emo

Test(s) of highest order unconditional interaction(s):
        R2-chng          F        df1        df2          p
X*W       ,0668     5,2289     1,0000    51,0000      ,0264
X*Z       ,0517     4,0468     1,0000    51,0000      ,0496
BOTH      ,1170     4,5798     2,0000    51,0000      ,0148
```

```
----------
    Focal predict: cond      (X)
          Mod var: met_cog   (W)
          Mod var: met_emo   (Z)

Conditional effects of the focal predictor at values of the moderator(s):

    met_cog    met_emo     Effect         se          t          p       LL
CI       ULCI
    16,8705     4,3434    -1,2634       ,4807    -2,6283      ,0113    -2,22
84     -,2984
    16,8705     5,5530     -,6513       ,3629    -1,7948      ,0786    -1,37
99      ,0772
    16,8705     6,7626     -,0393       ,4663     -,0842      ,9332     -,97
55      ,8969
    19,6140     4,3434     -,6682       ,3998    -1,6714      ,1008    -1,47
08      ,1344
    19,6140     5,5530     -,0562       ,2500     -,2247      ,8231     -,55
80      ,4457
    19,6140     6,7626      ,5559       ,3877     1,4339      ,1577     -,22
24     1,3342
    22,3576     4,3434     -,0730       ,4734     -,1543      ,8780    -1,02
34      ,8773
    22,3576     5,5530      ,5390       ,3589     1,5020      ,1393     -,18
14     1,2594
    22,3576     6,7626     1,1510       ,4675     2,4619      ,0172      ,21
24     2,0897

Data for visualizing the conditional effect of the focal predictor:
Paste text below into a SPSS syntax window and execute to produce plot.

DATA LIST FREE/
   cond       met_cog    met_emo    sba_sb     .
BEGIN DATA.
      ,0000    16,8705     4,3434     5,6314
     1,0000    16,8705     4,3434     4,3681
      ,0000    16,8705     5,5530     5,5952
     1,0000    16,8705     5,5530     4,9439
      ,0000    16,8705     6,7626     5,5590
     1,0000    16,8705     6,7626     5,5197
      ,0000    19,6140     4,3434     5,9503
     1,0000    19,6140     4,3434     5,2821
      ,0000    19,6140     5,5530     5,9141
     1,0000    19,6140     5,5530     5,8579
      ,0000    19,6140     6,7626     5,8779
```

```
     1,0000    19,6140     6,7626     6,4338
      ,0000    22,3576     4,3434     6,2693
     1,0000    22,3576     4,3434     6,1962
      ,0000    22,3576     5,5530     6,2330
     1,0000    22,3576     5,5530     6,7720
      ,0000    22,3576     6,7626     6,1968
     1,0000    22,3576     6,7626     7,3478
END DATA.
GRAPH/SCATTERPLOT=
 met_cog  WITH     sba_sb   BY       cond     /PANEL   ROWVAR=  met_emo  .

*********************** ANALYSIS NOTES AND ERRORS ************************

Level of confidence for all confidence intervals in output:
  95,0000

W values in conditional tables are the mean and +/- SD from the mean.

Z values in conditional tables are the mean and +/- SD from the mean.

------ END MATRIX -----
```

# Anxious appearance SAD

Run MATRIX procedure:

```
***************** PROCESS Procedure for SPSS Version 3.5 *****************

          Written by Andrew F. Hayes, Ph.D.       www.afhayes.com
    Documentation available in Hayes (2018). www.guilford.com/p/hayes3

**************************************************************************
Model  : 2
    Y  : sba_anx
    X  : cond
    W  : met_cog
    Z  : met_emo

Sample
Size:  57

**************************************************************************
OUTCOME VARIABLE:
 sba_anx

Model Summary
          R       R-sq        MSE          F        df1        df2
 p
      ,3970      ,1576     1,5592     1,9080     5,0000    51,0000      ,10
92

Model
              coeff         se          t          p       LLCI       ULCI
constant     3,8507     1,6666     2,3105      ,0249      ,5049     7,1964
cond         5,9681     3,1303     1,9065      ,0622     -,3164    12,2526
met_cog       ,0237      ,0784      ,3026      ,7634     -,1337      ,1811
Int_1        -,2831      ,1258    -2,2509      ,0287     -,5356     -,0306
met_emo      -,0878      ,1582     -,5551      ,5813     -,4055      ,2299
Int_2        -,1498      ,3335     -,4493      ,6551     -,8193      ,5197

Product terms key:
 Int_1    :        cond     x        met_cog
 Int_2    :        cond     x        met_emo

Test(s) of highest order unconditional interaction(s):
        R2-chng          F        df1        df2          p
X*W       ,0837     5,0664     1,0000    51,0000      ,0287
X*Z       ,0033      ,2018     1,0000    51,0000      ,6551
BOTH      ,0866     2,6216     2,0000    51,0000      ,0825
```

```
----------
    Focal predict: cond      (X)
          Mod var: met_cog   (W)
          Mod var: met_emo   (Z)

Conditional effects of the focal predictor at values of the moderator(s):

    met_cog    met_emo     Effect         se          t          p       LL
CI       ULCI
    16,8705     4,3434      ,5411      ,6373      ,8490      ,3999     -,73
84     1,8205
    16,8705     5,5530      ,3598      ,4811      ,7478      ,4580     -,60
61     1,3258
    16,8705     6,7626      ,1786      ,6183      ,2889      ,7739    -1,06
27     1,4199
    19,6140     4,3434     -,2357      ,5301     -,4446      ,6585    -1,29
98      ,8285
    19,6140     5,5530     -,4169      ,3314    -1,2579      ,2142    -1,08
23      ,2485
    19,6140     6,7626     -,5981      ,5140    -1,1637      ,2500    -1,63
00      ,4338
    22,3576     4,3434    -1,0124      ,6276    -1,6131      ,1129    -2,27
24      ,2476
    22,3576     5,5530    -1,1936      ,4758    -2,5088      ,0153    -2,14
88     -,2384
    22,3576     6,7626    -1,3749      ,6199    -2,2179      ,0310    -2,61
94     -,1304

Data for visualizing the conditional effect of the focal predictor:
Paste text below into a SPSS syntax window and execute to produce plot.

DATA LIST FREE/
   cond       met_cog    met_emo    sba_anx    .
BEGIN DATA.
      ,0000    16,8705     4,3434     3,8694
     1,0000    16,8705     4,3434     4,4105
      ,0000    16,8705     5,5530     3,7632
     1,0000    16,8705     5,5530     4,1230
      ,0000    16,8705     6,7626     3,6569
     1,0000    16,8705     6,7626     3,8355
      ,0000    19,6140     4,3434     3,9345
     1,0000    19,6140     4,3434     3,6988
      ,0000    19,6140     5,5530     3,8283
     1,0000    19,6140     5,5530     3,4114
      ,0000    19,6140     6,7626     3,7220
```

```
     1,0000    19,6140     6,7626     3,1239
      ,0000    22,3576     4,3434     3,9996
     1,0000    22,3576     4,3434     2,9872
      ,0000    22,3576     5,5530     3,8934
     1,0000    22,3576     5,5530     2,6997
      ,0000    22,3576     6,7626     3,7871
     1,0000    22,3576     6,7626     2,4122
END DATA.
GRAPH/SCATTERPLOT=
 met_cog  WITH     sba_anx  BY       cond     /PANEL   ROWVAR=  met_emo  .

********************** ANALYSIS NOTES AND ERRORS ************************

Level of confidence for all confidence intervals in output:
  95,0000

W values in conditional tables are the mean and +/- SD from the mean.

Z values in conditional tables are the mean and +/- SD from the mean.

------ END MATRIX -----
```

# Social behavior HC

Run MATRIX procedure:

```
***************** PROCESS Procedure for SPSS Version 3.5 *****************

          Written by Andrew F. Hayes, Ph.D.       www.afhayes.com
    Documentation available in Hayes (2018). www.guilford.com/p/hayes3

**************************************************************************
Model  : 2
    Y  : sba_sb
    X  : cond
    W  : met_cog
    Z  : met_emo

Sample
Size:  50

**************************************************************************
OUTCOME VARIABLE:
 sba_sb

Model Summary
          R       R-sq        MSE          F        df1        df2
 p
      ,3313      ,1098     1,2629     1,0851     5,0000    44,0000      ,38
19

Model
              coeff         se          t          p       LLCI       ULCI
constant     4,1987     1,2894     3,2563      ,0022     1,6001     6,7973
cond         1,4425     1,9724      ,7313      ,4684    -2,5326     5,4176
met_cog       ,0983      ,0684     1,4376      ,1576     -,0395      ,2362
Int_1        -,0531      ,0958     -,5539      ,5824     -,2461      ,1400
met_emo       ,1107      ,1935      ,5721      ,5702     -,2792      ,5006
Int_2        -,0358      ,2706     -,1323      ,8953     -,5811      ,5095

Product terms key:
 Int_1    :        cond     x        met_cog
 Int_2    :        cond     x        met_emo

Test(s) of highest order unconditional interaction(s):
        R2-chng          F        df1        df2          p
X*W       ,0062      ,3068     1,0000    44,0000      ,5824
X*Z       ,0004      ,0175     1,0000    44,0000      ,8953
BOTH      ,0090      ,2230     2,0000    44,0000      ,8010
```

```
----------
    Focal predict: cond     (X)
          Mod var: met_cog  (W)
          Mod var: met_emo  (Z)

Data for visualizing the conditional effect of the focal predictor:
Paste text below into a SPSS syntax window and execute to produce plot.

DATA LIST FREE/
   cond       met_cog    met_emo    sba_sb     .
BEGIN DATA.
      ,0000    16,1355     4,5224     6,2857
     1,0000    16,1355     4,5224     6,7103
      ,0000    16,1355     5,8181     6,4291
     1,0000    16,1355     5,8181     6,8073
      ,0000    16,1355     7,1137     6,5725
     1,0000    16,1355     7,1137     6,9043
      ,0000    19,8000     4,5224     6,6460
     1,0000    19,8000     4,5224     6,8762
      ,0000    19,8000     5,8181     6,7894
     1,0000    19,8000     5,8181     6,9732
      ,0000    19,8000     7,1137     6,9328
     1,0000    19,8000     7,1137     7,0702
      ,0000    23,4645     4,5224     7,0063
     1,0000    23,4645     4,5224     7,0421
      ,0000    23,4645     5,8181     7,1497
     1,0000    23,4645     5,8181     7,1391
      ,0000    23,4645     7,1137     7,2931
     1,0000    23,4645     7,1137     7,2361
END DATA.
GRAPH/SCATTERPLOT=
 met_cog  WITH     sba_sb   BY       cond     /PANEL   ROWVAR=  met_emo  .

********************** ANALYSIS NOTES AND ERRORS ************************

Level of confidence for all confidence intervals in output:
  95,0000

------ END MATRIX -----
```

# Anxious appearance HC

Run MATRIX procedure:

***************** PROCESS Procedure for SPSS Version 3.5 *****************

Written by Andrew F. Hayes, Ph.D. www.afhayes.com
Documentation available in Hayes (2018). www.guilford.com/p/hayes3

**************************************************************************

Model : 2
Y : sba_anx
X : cond
W : met_cog
Z : met_emo

Sample
Size: 50

**************************************************************************

OUTCOME VARIABLE:
sba_anx

Model Summary

| R | R-sq | MSE | F | df1 | df2 | p |
|---|---|---|---|---|---|---|
| ,3433 | ,1179 | 1,4155 | 1,1760 | 5,0000 | 44,0000 | ,3362 |

Model

| | coeff | se | t | p | LLCI | ULCI |
|---|---|---|---|---|---|---|
| constant | 5,3782 | 1,3651 | 3,9398 | ,0003 | 2,6270 | 8,1294 |
| cond | -1,3590 | 2,0882 | -,6508 | ,5185 | -5,5675 | 2,8495 |
| met_cog | -,0675 | ,0724 | -,9326 | ,3561 | -,2135 | ,0784 |
| Int_1 | ,0164 | ,1014 | ,1616 | ,8723 | -,1880 | ,2207 |
| met_emo | -,2439 | ,2048 | -1,1910 | ,2401 | -,6568 | ,1689 |
| Int_2 | ,2385 | ,2865 | ,8324 | ,4097 | -,3389 | ,8158 |

Product terms key:

| | | | | |
|---|---|---|---|---|
| Int_1 | : | cond | x | met_cog |
| Int_2 | : | cond | x | met_emo |

Test(s) of highest order unconditional interaction(s):

| | R2-chng | F | df1 | df2 | p |
|---|---|---|---|---|---|
| X*W | ,0005 | ,0261 | 1,0000 | 44,0000 | ,8723 |
| X*Z | ,0139 | ,6930 | 1,0000 | 44,0000 | ,4097 |
| BOTH | ,0193 | ,4819 | 2,0000 | 44,0000 | ,6208 |

```
----------
    Focal predict: cond      (X)
          Mod var: met_cog   (W)
          Mod var: met_emo   (Z)

Data for visualizing the conditional effect of the focal predictor:
Paste text below into a SPSS syntax window and execute to produce plot.

DATA LIST FREE/
   cond       met_cog    met_emo    sba_anx    .
BEGIN DATA.
      ,0000    16,1355     4,5224     3,1854
     1,0000    16,1355     4,5224     3,1693
      ,0000    16,1355     5,8181     2,8694
     1,0000    16,1355     5,8181     3,1622
      ,0000    16,1355     7,1137     2,5533
     1,0000    16,1355     7,1137     3,1550
      ,0000    19,8000     4,5224     2,9380
     1,0000    19,8000     4,5224     2,9819
      ,0000    19,8000     5,8181     2,6219
     1,0000    19,8000     5,8181     2,9748
      ,0000    19,8000     7,1137     2,3059
     1,0000    19,8000     7,1137     2,9677
      ,0000    23,4645     4,5224     2,6906
     1,0000    23,4645     4,5224     2,7945
      ,0000    23,4645     5,8181     2,3745
     1,0000    23,4645     5,8181     2,7874
      ,0000    23,4645     7,1137     2,0584
     1,0000    23,4645     7,1137     2,7803
END DATA.
GRAPH/SCATTERPLOT=
 met_cog  WITH     sba_anx  BY       cond     /PANEL   ROWVAR=  met_emo  .

********************** ANALYSIS NOTES AND ERRORS ************************

Level of confidence for all confidence intervals in output:
  95,0000

------ END MATRIX -----
```
